# Supplementary material for: The 5S RNP Couples p53 Homeostasis to Ribosome Biogenesis and Nucleolar Stress
Source: Cell Rep. 2013 Oct 17;5(1):237–47. doi: 10.1016/j.celrep.2013.08.049 (PMC3808153; doi:10.1016/j.celrep.2013.08.049)
Supplement: Document S1. Tables S1–S3 [file mmc1.pdf]

Table S1. List of siRNAs used in RNAi experiments. Related to Figures 1, 3, 4, 5, S1, S3 and S4.

**Target**	**Sequence (5'→3')**	**Source**
Control	CGUACGCGGAAUACUUCGATT	(Elbashir et al., 2002)
RPL5	UACUUUAAGAGAUACCAAGTT	(Kuroda et al., 2011)
RPL11	GGUGCGGGAGUAUGAGUUA	(Bursac et al., 2012)
RRS1	AUCCGGACACCAGAGUAA	(Gambe et al., 2009)
BXDC1	GGGAAGAUUUACUUUCGAA	Dharmacon SMARTpool
	UGGCGAUGAUUUCGAUGUA	
	UGAGAAGAGAGAACCGAAA	
	UUGCAGAACACCACGGAUU	
PICT1	GAGACCGGUUCAAGAGCUU	(Lee et al., 2012)
NOP2	CACCUGUUCUAUCACAGUATT	D Lafontaine, personal communication
	GCAACGAUCACCUAAAUUATT	
PAK1IP1	CUAGUGUGCCUCUGCGAAU	Dharmacon SMARTpool
	UUUAAUCAGUGGAGCGGAA	
	CAUCACAGUGGUACAAUAA	
	GUCGGUUGGUACAGAUAAA	
BOP1	AUGGCAUGGUGUACAAUGATT	(Rohrmoser et al., 2007)
5S	GGGAAUACCGGGUGCUGUA	(Li and Gu 2011)
	AGUACUUGGAUGGGAGACC	
TFIIIA	AACAUUUGAUUCCUUAUCATT	Qiagen Genepool
	CAGCAUACCAAUGAACCUCUA	

Table S2. List of Oligonucleotide probes used in Northern Blotting. Related to Figures 1, S1 and S2.

**Target**	**Sequence (5'→3')**
**5S**	CCGAGATCAGACGAGATCGGGCGCGTTCAGGGTGGTATGG
**5.8S**	CAATGTGTCCTGCAATTCAC

Table S3. List of antibodies used in Western blotting. Related to Figures 1, 5, S1, S2, S3 and S4.

**Target/Antibody**	**Source**
RPL5	Bethyl (A303-933A)
RPL11	Abcam (ab79352)
RRS1	Santa Cruz (sc-87412)
BXDC1	Santa Cruz (sc-81060)
PICT1	Santa Cruz (sc-46615)
NOP2	Sigma (**HPA040119)**
PAK1IP1	Bethyl (A301-550A)
RBM28	Santa Cruz (sc-102075)
TFIIIA	Bethyl (A303-621A)
p21	Santa Cruz (sc-397)
p53	Bethyl (A300-248A)
$p14^{ARF}$	Calbichem (NA70)
CSL4	Watkins lab (Sloan et al., 2013)
XRN2	Bethyl (A301-103A)
Fibrillarin	Santa Cruz (sc-25397)
FLAG	Sigma (M2)
