# Supplementary material for: The 5S RNP Couples p53 Homeostasis to Ribosome Biogenesis and Nucleolar Stress
Source: Cell Rep. 2013 Oct 17;5(1):237–47. doi: 10.1016/j.celrep.2013.08.049 (PMC3808153; doi:10.1016/j.celrep.2013.08.049)
Supplement: Document S2. Article plus Supplemental Information [file mmc2.pdf]

Cell Reports
Article

Cell PRESS Open ACCESS

The 5S RNP Couples p53 Homeostasis to Ribosome Biogenesis and Nucleolar Stress

Katherine E. Sloan,[1,2] Markus T. Bohnsack,[2] and Nicholas J. Watkins[1,*]
[1]ICaMB, Newcastle University, Newcastle upon Tyne NE2 4HH, UK
[2]Centre for Biochemistry and Molecular Cell Biology, Institute for Molecular Biology, Medical Faculty, Georg-August University, 37073 Goettingen, Germany
*Correspondence: n.j.watkins@ncl.ac.uk
http://dx.doi.org/10.1016/j.celrep.2013.08.049
This is an open-access article distributed under the terms of the Creative Commons Attribution License, which permits unrestricted use, distribution, and reproduction in any medium, provided the original author and source are credited.

SUMMARY

Several proto-oncogenes and tumor suppressors regulate the production of ribosomes. Ribosome biogenesis is a major consumer of cellular energy, and defects result in p53 activation via repression of mouse double minute 2 (MDM2) homolog by the ribosomal proteins RPL5 and RPL11. Here, we report that RPL5 and RPL11 regulate p53 from the context of a ribosomal subcomplex, the 5S ribonucleoprotein particle (RNP). We provide evidence that the third component of this complex, the 5S rRNA, is critical for p53 regulation. In addition, we show that the 5S RNP is essential for the activation of p53 by p14^{ARF}, a protein that is activated by oncogene overexpression. Our data show that the abundance of the 5S RNP, and therefore p53 levels, is determined by factors regulating 5S complex formation and ribosome integration, including the tumor suppressor PICT1. The 5S RNP therefore emerges as the critical coordinator of signaling pathways that couple cell proliferation with ribosome production.

INTRODUCTION

The production of eukaryotic ribosomes is a major consumer of cellular energy and regulated by several tumor suppressors and proto-oncogenes (Stumpf and Ruggero, 2011). Indeed, ribosome biogenesis is upregulated by the oncogene *c-Myc*, downregulated by the tumor suppressor p14^{ARF}, and is linked to the regulation of the tumor suppressor p53 (Stumpf and Ruggero, 2011). Several genetic diseases, such as Diamond-Blackfan anemia, dyskeratosis congenita, and Treacher Collins syndrome, arise due to defects in ribosome production, and in a number of cases, this has been linked to the misregulation of p53 (Freed et al., 2010; Fumagalli and Thomas, 2011; Narla and Ebert, 2010). Surprisingly, several of these diseases, which are known as ribosomopathies, also predispose patients to a range of cancers.

The tumor suppressor p53 is activated by a wide range of cellular stresses, leading to either repair of the cellular damage, cell-cycle arrest, apoptosis, or senescence. A key regulator of p53 is mouse double minute 2 homolog (MDM2), an E3 ubiquitin ligase that inhibits p53 activity through proteasome-mediated degradation. Several ribosomal proteins (RPs) bind to and inactivate MDM2, thereby activating p53 (Chakraborty et al., 2011), but recent work has shown that only RPL5 and RPL11 are essential for p53 activation in response to a block in ribosome biogenesis (Bursać et al., 2012; Fumagalli et al., 2012; Sun et al., 2010). MDM2 mutations found in several cancers, which disrupt the RPL11-MDM2 interaction, attenuate the p53-mediated response to nucleolar/ribotoxic stress and accelerate c-Myc-induced lymphomagenesis in a mouse model system (Macias et al., 2010; Pan et al., 2011). RPL11 also binds to and promotes the activity of the tumor suppressor p14^{ARF} (Dai et al., 2012), which interacts with and represses MDM2 and is activated by the overexpression of oncogenes such as *c-Myc*.

Although RPL5 and RPL11 inhibit MDM2 outside the ribosome, it is unlikely that they perform this function individually, as free ribosomal proteins are unstable in mammalian cells (Lam et al., 2007). RPL11, together with RPL5 and the 5S rRNA, comprise the 5S ribonucleoprotein particle (RNP), an essential subcomplex of the large ribosomal subunit. RPL5 binds the 5S rRNA and the 5S rRNA/RPL5 complex and then localizes to the nucleolus, where it binds RPL11 and is integrated into the ribosome (Chakraborty et al., 2011). RPL5 and RPL11 have been shown to be mutually dependent on one another for stability/accumulation when ribosome biogenesis is blocked (Bursać et al., 2012). Furthermore, it has been demonstrated that RPL11 activates p53 cooperatively with RPL5 and mutations, which are predicted to impede RPL11 interaction with the 5S rRNA, inhibit this induction (Horn and Vousden, 2008).

Proteins that regulate 5S RNP formation, localization, and integration into the ribosome are predicted to be central in regulating MDM2 activity and, therefore, p53 levels in the cell. PICT1 (GLTSCR2) has recently been identified as a novel tumor suppressor that induces p53 and activates the PTEN pathway/ATM checkpoint in response to DNA damage (Kim et al., 2011). Interestingly, PICT1 has also been shown to retain RPL11 in the nucleolus in normal cells. However, under ribotoxic stress conditions, RPL11 and PICT1 relocalize to the nucleoplasm, where they activate p53 (Sasaki et al., 2011). Mechanistic details on how PICT1 performs this function are currently

CrossMark

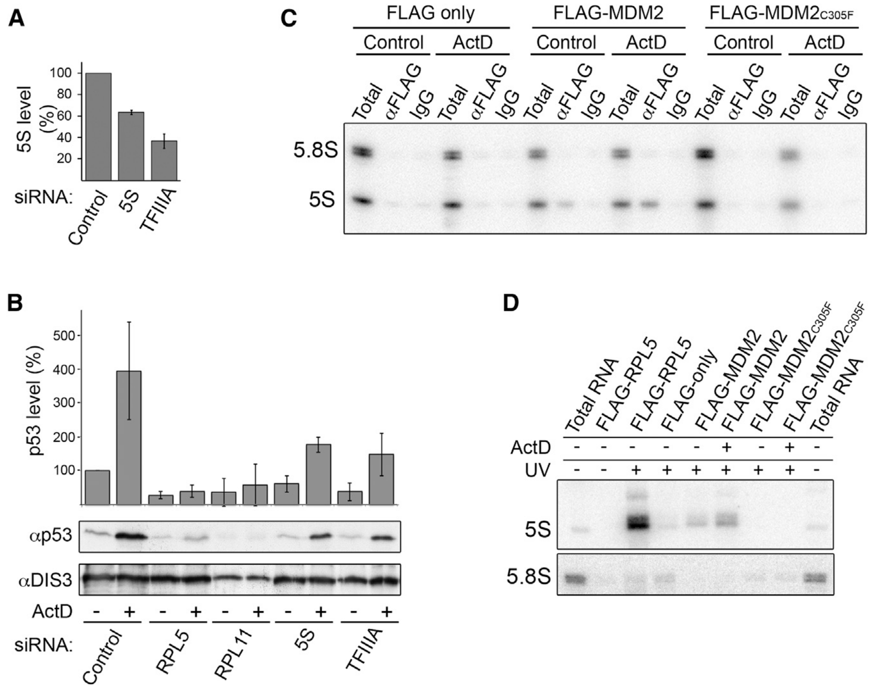

Figure 1. The 5S RNP Complex Is Required for p53 Induction through Direct Interaction with MDM2 in Response to Ribotoxic Stress

(A) The level of newly synthesized 5S rRNA in cells transfected with control siRNAs or those targeting TFIIIA or the 5S rRNA itself was determined by pulse-labeling followed by gel electrophoresis. Quantification is based on three independent experiments. Error bars indicate SD.

(B) U2OS cells were transfected with siRNAs against the core 5S RNP components and either untreated (–) or treated (+) with ActD for 10 hr. p53 levels were analyzed by western blotting. Quantification is based on three independent experiments. Error bars indicate SD.

(C) Anti-FLAG immunoprecipitations of extracts from HEK293 cells expressing FLAG-MDM2, -MDM2$_{C305F}$, or the FLAG-tag. Coprecipitated 5S and 5.8S rRNAs were detected by northern blotting. IgG, immunoglobulin G.

(D) Cells expressing FLAG-tagged proteins were UV crosslinked, and covalently linked RNA protein complexes were purified. Coprecipitated 5S and 5.8S rRNAs were detected by northern blotting.

See also Tables S1, S2, and S3 and Figure S1.

lacking, but because this protein is in fact homologous to the yeast ribosome biogenesis factor Nop53, we hypothesize that it may activate p53 through a role in ribosome biogenesis. Several other factors have been linked to the formation of the 5S RNP and its integration into the ribosome in yeast, making these good candidates for performing this role, but their human counterparts are yet to be characterized (Talkish et al., 2012; Zhang et al., 2007). Furthermore, how the function of RPL11 and RPL5 in p53 signaling relates to their role in ribosome production also remains unclear at present, but it is exciting to speculate that their dual function reflects a coordinated pathway coupling ribosome biogenesis to cell proliferation.

Given the importance of RPL11 and RPL5 as components of the p53 signaling pathway (Chakraborty et al., 2011), 5S RNP biology is an underinvestigated area of research that is relevant for understanding the basis of many human ribosomopathies. We show that the 5S rRNA, as part of an assembled 5S RNP complex, is essential for p53 homeostasis and p53 activation when p14^{ARF} is expressed. Our data indicate that the 5S RNP functions as a central regulator of p53, providing a means to directly relate ribosome biogenesis to cellular growth and cell-cycle regulation.

RESULTS

The 5S rRNA, Together with RPL11 and RPL5, Is Required for p53 Homeostasis and the Activation of p53 when Ribosome Biogenesis Is Inhibited

MDM2 is a critical negative regulator of p53 levels and the ribosomal proteins RPL5 and RPL11 have been shown to inhibit MDM2 activity when ribosome biogenesis is blocked, leading to p53 activation. During ribosome biogenesis, RPL5 and RPL11 both associate with the 5S rRNA to form the 5S RNP, an essential subcomplex of the ribosome. To investigate the importance of the 5S rRNA in regulating MDM2/p53, two parallel approaches were taken to decrease the production of the 5S rRNA in U2OS cells and to monitor the effect on p53 levels: the 5S rRNA-specific transcription factor IIIA (TFIIIA) was depleted by RNAi and the 5S rRNA itself was directly targeted using siRNAs (Li and Gu, 2011). The effect of depleting RPL5 and RPL11 by RNAi on p53 levels was also analyzed for comparison (Figure 1 and Figure S1A). Decreasing TFIIIA to <5% of the normal level (Figure S1A) resulted in an approximately 3-fold reduction in 5S rRNA production in pulse-labeling experiments, whereas direct targeting of the 5S rRNA reduced 5S rRNA synthesis to about 60% of that seen in control cells (Figure 1A).

If components of the 5S RNP are required for p53 activation when ribosome production is blocked, depletion of these factors should diminish p53 induction by the chemotherapeutic agent Actinomycin D (ActD), which blocks rRNA synthesis by inhibiting RNA polymerase I. Interestingly, knockdown of RPL11, RPL5, TFIIIA, or the 5S rRNA all resulted in a reduction in the basal levels of p53 in untreated U2OS cells, suggesting that these 5S RNP components are required for maintaining cellular p53 levels (Figure 1B). In addition, treatment of cells with ActD caused the accumulation of p53 (approximately 4-fold), but reducing the levels of RPL5 or RPL11 eradicated this induction. Decreasing 5S rRNA levels also reduced but did not abolish ActD-mediated p53 activation (Figure 1B). This is consistent with the fact that 5S rRNA synthesis was only diminished and not eradicated (Figure 1A). Taken together, our data show that the 5S rRNA, together with RPL5 and RPL11, is required for ActD induction of p53 and p53 homeostasis in normal cells.

Cell PRESS Open ACCESS

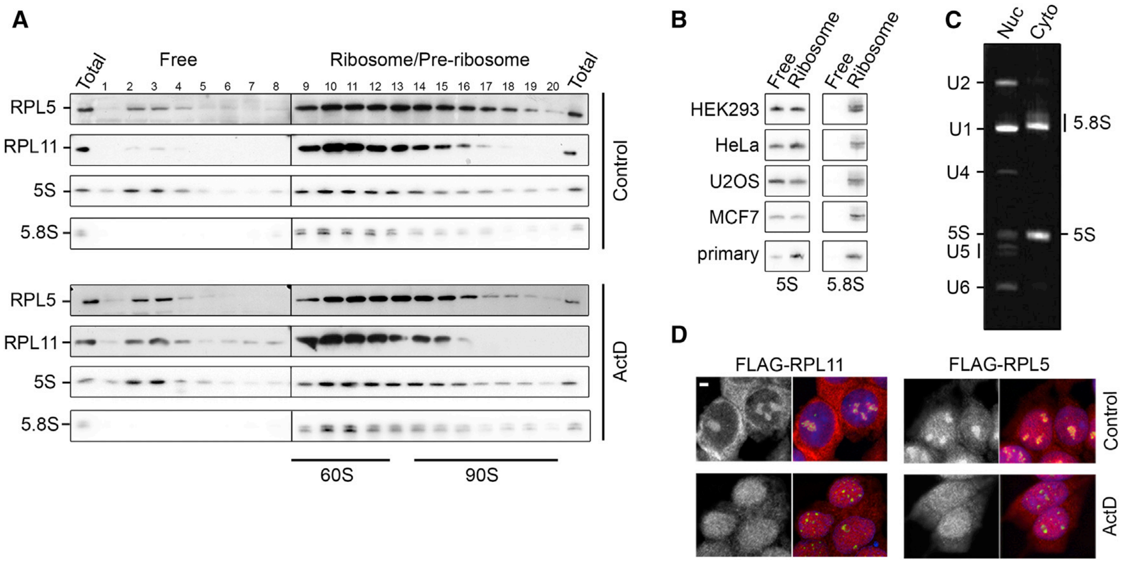

Figure 2. A 5S RNP Complex Containing 5S rRNA, RPL5, and RPL11 Only Accumulates in the Nucleoplasm when Ribosome Production Is Inhibited

(A) Extracts from HEK293 cells either treated (+) or untreated (−) with ActD were analyzed by glycerol gradient centrifugation followed by western and northern blotting. Antibodies or probes used are indicated to the left of each panel.

(B) Levels of 5S and 5.8S rRNAs in free (pooled fractions 1–7) and ribosomal (pooled fractions 9–20) complexes in various cell lines were analyzed by northern blotting. Primary, primary dermal fibroblasts.

(C) RNA from HeLa cell nuclear and cytoplasmic extracts was separated by polyacrylamide gel electrophoresis and detected using ethidium bromide staining.

(D) The localization of newly synthesized FLAG-RPL5 or -RPL11 in HEK293 stable cell lines was determined by immunofluorescence. Cells were grown in the presence (ActD) or absence (control) of ActD, and antibodies against the FLAG-tag (red/greyscale) and fibrillarin (green; nucleolar marker) were used. Nuclear material was detected by DAPI staining (blue). The scale bar represents 1 μm.

See also Tables S2 and S3 and Figure S2.

MDM2 Directly Contacts the 5S rRNA

RPL11 and RPL5 activate p53 by binding to and inhibiting MDM2 (Chakraborty et al., 2011). We next asked whether the 5S rRNA is also specifically associated with MDM2. For this, human embryonic kidney 293 (HEK293) cells stably expressing inducible FLAG-MDM2 and an MDM2 mutant (C305F), which is not able to interact with RPL5 and RPL11 (Macias et al., 2010), were generated (Figure S1B). Immunoprecipitation experiments using whole cell extracts revealed that FLAG-MDM2 was associated with the 5S rRNA, but not the 5.8S rRNA (Figure 1C). Importantly, the amount of 5S rRNA coprecipitated from extracts was significantly higher after cells were treated with ActD. Neither 5S rRNA nor 5.8S rRNA coprecipitated with FLAG-MDM2$_{C305F}$ indicating that the 5S rRNA-MDM2 association is dependent on the binding of RPL11 and RPL5 to MDM2. Importantly, the specific association of MDM2 with the 5S rRNA was also observed by immunoprecipitation using MDM2-specific antibodies from U2OS cell extracts (Figure S1C).

MDM2 has been demonstrated to bind both RPL11 and RPL5 raising the possibility that MDM2, which has previously been shown to bind RNA (Elenbaas et al., 1996), may also directly contact the 5S rRNA. To test this, cells expressing FLAG-tagged MDM2, MDM2$_{C305F}$, RPL5, or the FLAG tag alone (Figure S1B) were irradiated with UV light. Cells expressing FLAG-MDM2 or MDM2$_{C305F}$ that had been treated with ActD were also included. The proteins, and any covalently bound RNAs, were then isolated from extracts derived from these cells using a denaturing two-step purification procedure, and the associated RNAs were analyzed by northern blotting. The 5S rRNA was found to crosslink strongly to RPL5, as expected, but also specifically to MDM2 (Figure 1D). Consistent with our immunoprecipitation data, the amount of 5S rRNA crosslinked to MDM2 increased when cells were treated with ActD and the MDM2$_{C305F}$ mutation abolished this interaction. These data demonstrate that, in addition to directly binding RPL11 and RPL5, MDM2 also directly contacts the 5S rRNA.

The 5S RNP, a Complex of RPL5, RPL11, and the 5S rRNA, Accumulates when Ribosome Biogenesis Is Blocked

Our data imply that, rather than functioning individually, the 5S rRNA, RPL11, and RPL5 act as a complex to activate p53. However, how these factors accumulate outside the ribosome in human cells, and how this is affected when ribosome biogenesis is blocked, is currently unknown. We used glycerol gradient centrifugation to define 5S RNP complexes that exist in normal cells and in cells in which ribosome production has been perturbed by treatment with ActD.

In untreated HEK293 cells, the 5S rRNA, RPL11, and RPL5 were present in complexes containing mature large ribosomal subunits (indicated by the position of the 5.8S rRNA; Figure 2A; fractions 9–18). These fractions also contained ribosome biogenesis factors, such as RBM28 (Figure S2A) and therefore preribosomal complexes. A significant proportion of RPL5 and the 5S rRNA were found in fractions 2–4 (Figure 2A) demonstrating

that they are naturally present in nonribosomal fractions. The relatively high level of free 5S rRNA was not specific to HEK293 cells but also observed in HeLa, U2OS, MCF7, and primary human fibroblast cells (Figure 2B). Interestingly, RPL11 only significantly accumulated in this free pool after the cells were treated with ActD (Figure 2A). Consistent with our earlier data identifying interactions between 5S RNP components and MDM2 (Figures 1C and 1D), FLAG-MDM2 expressed in HEK293 cells also comigrated with RPL5, RPL11, and the 5S rRNA in the free fractions of a glycerol gradient, but not in the ribosomal fractions (Figure S2B). Immunoprecipitation using extracts from stable cell lines expressing FLAG-tagged RPL5/RPL11 was used to confirm that the comigrating RPL5, RPL11, and 5S rRNA in fractions 2–4 form a stable complex (Figures S1B and S2C). Interestingly, none of the ribosome biogenesis factors analyzed, including RRS1 and BXDC1, which are associated with a free 5S RNP when ribosome biogenesis is blocked in yeast (Zhang et al., 2007), were present in the free fractions under any of the conditions tested (Figure S2A).

Taken together, these data indicate that the 5S rRNA, RPL5, and RPL11 components, together with MDM2, are present as a complex in the free fractions of the gradient and that the amount of RPL11 in this complex increases when ribosome biogenesis is blocked.

The 5S RNP Accumulates in the Nucleoplasm when Ribosome Production Is Inhibited

Our data demonstrate that a free 5S RNP accumulates when ribosome biogenesis is blocked. This complex interacts with MDM2, but it is not clear where this interaction takes place. RPL11 and RPL5 were predicted to repress MDM2 in the nucleoplasm (Chakraborty et al., 2011), but a recent report suggested that the RPL5/RPL11/MDM2 complex accumulates in the nucleolus when ribosome biogenesis is blocked (Bursać et al., 2012). We investigated the subcellular distribution of the 5S RNP. Analysis of the RNA content of nuclear and cytoplasmic extracts revealed a high level of the 5S rRNA in the nucleoplasm (Figure 2C). These nuclear extracts do not contain nucleolar material (Watkins et al., 2004) indicating that the 5S rRNA is present in the nucleoplasm at levels higher than the spliceosomal U5 snRNA (~200,000 copies/cell; Reddy and Busch, 1988).

To determine the localization of newly synthesized RPL11 and RPL5, we used immunofluorescence to analyze HEK293 cells expressing tetracycline-regulated FLAG-tagged RPL11 and RPL5, in the presence or absence of ActD to block ribosome production. Importantly, the FLAG-tagged proteins were not overexpressed and showed the same profile on glycerol gradients as the endogenous proteins (Figures S1B and S2B). In control cells, RPL11 was predominantly present in the cytoplasm and nucleolus, whereas RPL5 was found throughout the cell with significant levels present in the nucleolus and nucleoplasm (Figure 2D). After treatment with ActD, there was a significant increase in RPL11 levels in the nucleoplasm and a major decrease in the levels of both RPL5 and RPL11 in the nucleolus (Figure 2D). This suggests that active ribosome biogenesis is required for the nucleolar localization/retention of both RPL5 and RPL11.

Taken together, our data indicate that the 5S/RPL5 complex is naturally abundant in the nucleoplasm but that the 5S RNP (5S rRNA/RPL5/RPL11) accumulates in the nucleoplasm when ribosome biogenesis is blocked. Along with RPL5 and RPL11, the 5S rRNA interacts with MDM2, implying that the trimeric 5S RNP complex binds MDM2 in the nucleoplasm to affect p53 levels. Modulating the levels of the 5S RNP directly controls MDM2 activity. Consequently, the pathways that mediate 5S RNP formation, localization, and integration into the ribosome are central for regulating p53 levels and cellular proliferation.

Factors Linked to 5S RNP Biogenesis and p53 Activation through the RPL5/RPL11 Pathway Are Important for the Production of the Large Ribosomal Subunit

The nucleolar protein PICT1 has recently emerged as a key regulator of p53 and has been proposed to function by retaining RPL11 in the nucleolus (Sasaki et al., 2011). Similarly, the putative tumor suppressor PAK1IP1 is suggested to act through the ribosomal protein-MDM2 pathway, but it is currently unknown whether these factors function as regulators of the interaction of the 5S RNP with MDM2 or whether they perform these roles as components of the ribosome biogenesis machinery. We therefore investigated the role of several nucleolar proteins linked either to p53 regulation (PICT1 and PAK1IP1) or known to be important for 5S RNP integration into ribosomes in yeast (RRS1, BXDC1, and NOP2) in ribosome biogenesis in human cells. We also analyzed the importance of the 5S RNP components RPL5, RPL11, and the 5S rRNA for ribosome production. Individual proteins or the 5S rRNA were depleted using RNAi, and the production of newly synthesized ribosomes was determined by pulse-chase labeling of mature (18S, 28S, and 5.8S) and precursor (47S/45S and 32S) rRNAs (Figure 3A) followed by agarose-glyoxal or polyacrylamide gel electrophoresis (Figures 3B, S1A, and S3A).

Reducing the levels of RPL5, RPL11, PICT1, RRS1, or NOP2 resulted in a dramatic reduction in the production of the 28S and 5.8S rRNAs with minimal effects on 18S rRNA accumulation (Figure 3B). This is similar to the effect seen upon knockdown of the large ribosomal subunit biogenesis factor, BOP1 (Hölzel et al., 2005; Strezoska et al., 2000; Figure 3B). Knockdown of BXDC1, PAK1IP1, TFIIIA, or 5S rRNA also specifically decreased accumulation of the large subunit rRNAs, 28S and 5.8S, although to a lesser extent than with the other knockdowns. Interestingly, even though the siRNAs targeting TFIIIA and the 5S rRNA only had minimal effects on 5S rRNA synthesis (Figures 1A and 3B), this was sufficient to reduce 28S and 5.8S rRNA accumulation demonstrating that production/incorporation of the 5S RNP is required for large ribosomal subunit formation.

Whereas most of the knockdowns had a significant effect on 28S and 5.8S rRNA accumulation, their effect on 5S rRNA production was minimal implying that the accumulation of the 5S rRNA, which is transcribed by RNA pol III, occurs independently of the RNA pol I-transcribed rRNAs. Indeed, ActD inhibition of RNA pol I transcription or blocking pre-rRNA processing by treatment with 5-fluorouracil (5FU) had no notable effect on 5S rRNA accumulation (Figure 3C). Knockdown of RPL5, however, resulted in a significant reduction in 5S rRNA levels and the accumulation of a smear of longer 5S pre-rRNAs (Figure 3D). After transcription, the 5S rRNA undergoes 3′ processing to remove a 2 to 3 nt extension (Ciganda and Williams, 2011), and our

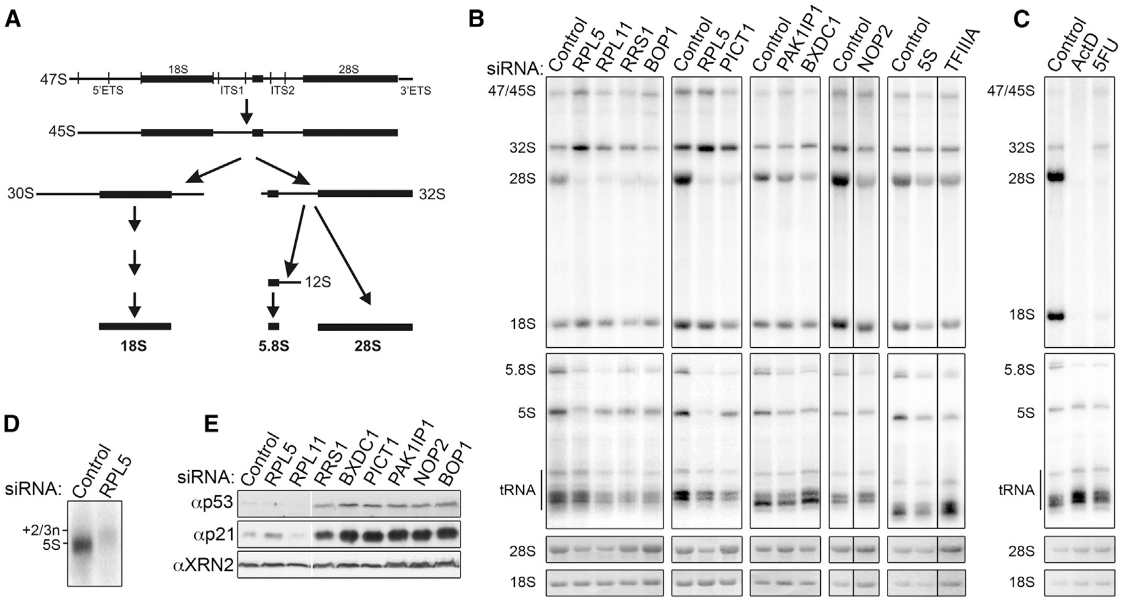

Figure 3. Putative 5S RNP Biogenesis Factors Are Required for Large Ribosomal Subunit Production and Their Depletion Activates p53

(A) Outline of the major pre-rRNA processing pathway in humans.

(B and C) HEK293 cells depleted of 5S RNP components or putative 5S biogenesis factors by RNAi, or treated with chemotherapeutics (ActD and 5FU), were pulse-labeled using ^{32}P orthophosphate. Labeled RNA was analyzed by agarose/acrylamide gel electrophoresis followed by phosphorimager analysis. Total RNA was visualized using ethidium bromide staining.

(D) Magnification of the pre-5S rRNA in cells depleted of RPL5 from (B).

(E) p53 levels in U2OS cells depleted of ribosome biogenesis factors were determined by western blotting. The antibodies used are indicated to the left of the panels.

See also Tables S1 and S3 and Figure S3.

data indicate that RPL5 is essential for this maturation as well as for 5S rRNA accumulation.

Depletion of a ribosome biogenesis factor would block ribosome production, leading to increased accumulation of the 5S RNP and therefore cause activation of p53. However, if a ribosome biogenesis factor was also required for MDM2 regulation, depleting this protein would have no effect on p53 levels in the cell. Indeed, knockdown of RPL11 or RPL5 did not increase the levels of p53 or its downstream target, p21, even though these knockdowns efficiently blocked large ribosomal subunit production (Figures 3B and 3E). In contrast, depleting BOP1, RRS1, BXDC1, PAK1IP1, PICT1, or NOP2 in U2OS cells resulted in significant increases in the levels of p53 and p21 (Figure 3E). Furthermore, p53 activation by the knockdown of factors such as PICT1 was nullified by the codepletion of RPL5 or RPL11 confirming that they function through the 5S RNP/MDM2 pathway (Figure S3B).

Taken together, our data demonstrate that knockdown of factors required for the synthesis of the large ribosomal subunit, other than components of the 5S RNP, results in the activation of p53.

RRS1 and BXDC1 Are Important for the Nucleolar Localization of the 5S RNP

Blocking ribosome biogenesis using ActD resulted in the accumulation of the 5S RNP in the nucleoplasm (Figure 2D). ActD causes a major change in the nucleolar structure, and we therefore wanted to test whether knockdown of individual ribosome biogenesis factors also leads to the relocalization of the 5S RNP. We therefore next examined whether any of the putative 5S RNP biogenesis factors that are essential for the production of the large ribosomal subunit are also required for the nucleolar localization of RPL5 and RPL11. To allow analysis of the localization of RPL5 and RPL11 produced only after the biogenesis factors were depleted, HEK293 cells expressing inducible FLAG-RPL5 or FLAG-RPL11 were transfected with siRNAs, and 36 hr later, expression of the tagged-protein was induced.

Consistent with our previous data (Figure 2D), RPL11 was predominantly found in the nucleolus (Figure 4; marker: fibrillarin) and cytoplasm of control cells and did not significantly accumulate in the nucleoplasm, whereas RPL5 localized throughout the cell. Knockdown of either RRS1 or BXDC1 resulted in a major reduction in the amount of RPL5 in the nucleolus (Figure 4). In contrast, only knockdown of BXDC1 affected the nucleolar localization of RPL11. However, a common feature of depletion of either RRS1 or BXDC1 was an increase in the nucleoplasmic accumulation of both RPL5 and RPL11. The knockdown of PICT1, PAK1IP1, NOP2, or BOP1 did not significantly affect the distribution of RPL5 in the cell. In contrast, depletion of any of these proteins resulted in an increased relocation of RPL11 from the cytoplasm to the nucleoplasm but no significant change in the amount present in the nucleolus. Surprisingly, depletion of TFIIIA did not alter the localization of either RPL5 or RPL11, although this knockdown had a similar effect on rRNA biogenesis

Cell PRESS
Open ACCESS

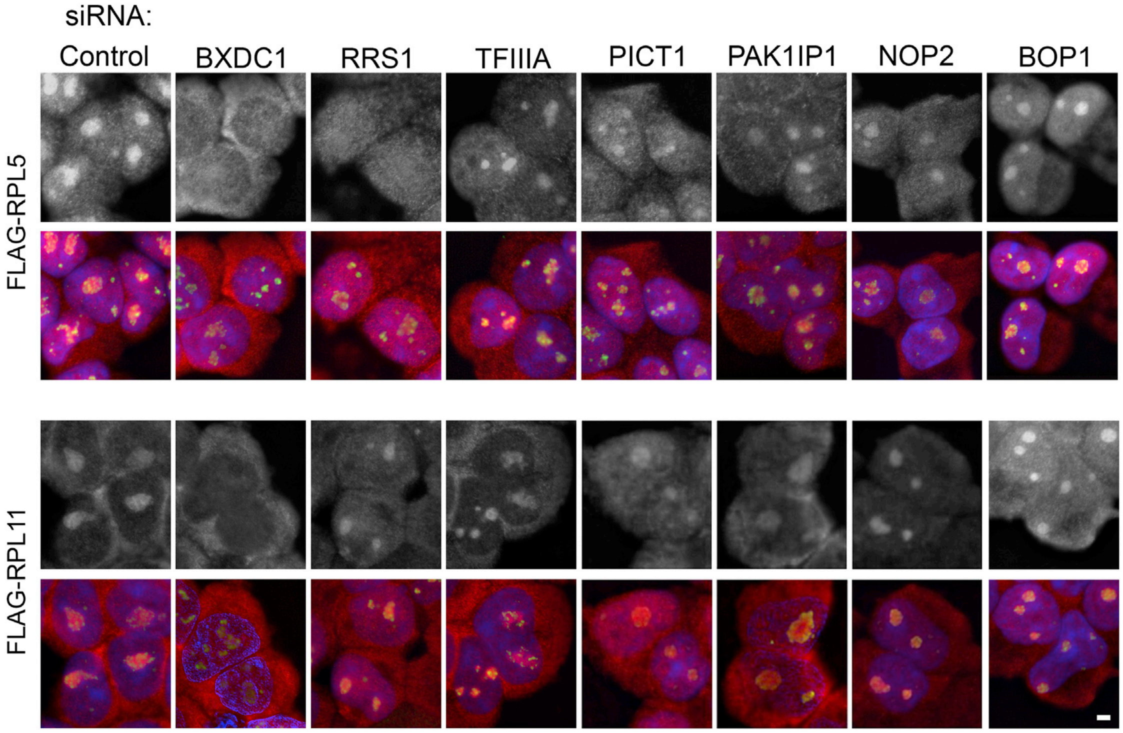

Figure 4. The Biogenesis Factors RRS1 and BXDC1 Are Required for Nucleolar Localization of the 5S RNP
Inducible HEK293 FLAG-RPL5 or FLAG-RPL11 cells were grown on coverslips and depleted of various 5S/ribosome biogenesis factors using RNAi. For the final 12 hr of the knockdown, tagged protein expression was induced and cells were fixed. Immunofluorescence was performed using antibodies to detect the FLAG-tag (red/greyscale) and fibrillarin (green). Nuclear material was visualized by DAPI staining (blue). The scale bar represents 1 μm.
See also Table S1.

to depleting PAK1IP1 (Figures 3B and 4). We conclude that, if the 5S rRNA is essential for RPL5 and RPL11 accumulation, it is likely that knockdown of TFIIIA results simply in reduced levels of the 5S RNP and does not cause its mislocalization.

Our data therefore indicate that, with the exception of depleting TFIIIA, blocking large ribosomal subunit production results in an increase in the nucleoplasmic levels of RPL11. Interestingly, RRS1 and BXDC1 are important for the nucleolar localization of RPL11 and RPL5, despite the fact that these proteins are not associated with a nonribosomal 5S RNP complex as they are in yeast (Zhang et al., 2007).

5S rRNA Integration into the Ribosome Is Slow

The amount of free 5S RNP in the cell must be strictly controlled in order to maintain appropriate levels of p53 and to regulate the rate of proliferation. The abundance of the free 5S RNP is influenced by the rate of both 5S RNP synthesis and its integration into the ribosome. Therefore, identification of factors that mediate 5S RNP integration into the ribosome will be critical for discovering key regulators of p53 in this pathway. We developed an assay with which 5S RNP recruitment into ribosomes could be monitored. For this, HEK293 cells were pulse-labeled with ^{32}P orthophosphate and then incubated with normal media for various time points over a 12 hr period. Extracts prepared from these cells were then analyzed by glycerol gradient centrifugation followed by gel electrophoresis (Figures 5A and S4A). As observed for the steady-state 5S rRNA (Figure 2A), the newly synthesized 5S rRNA was found in two pools: the ribosomal and preribosomal complexes (fractions 9–20) and a free pool (fractions 1–7). The percentage of the 5S rRNA in the free and ribosomal/preribosomal complexes was then calculated and plotted relative to the processing of the 28S rRNA (Figure 5B).

Integration of the newly transcribed 5S rRNA into the ribosome fractions was slow, with more than 40% of the 5S rRNA found in the free pool after 6 hr and more than 30% still free after 12 hr. In contrast, processing of the RNA-pol-I-transcribed precursor-rRNAs into mature rRNAs, as measured by the accumulation of 28S rRNA, was 90% complete after 6 hr. The slow recruitment of newly synthesized 5S rRNA into the ribosome is probably due to the 5S rRNA being recruited from the large pool of free 5S rRNA naturally present in the nucleus (see Figure 2).

PICT1 and RPL11 Are Required for 5S RNP Integration into the Ribosome

Using this recruitment assay, we then established which of the potential 5S RNP biogenesis factors are required for 5S RNP integration into the ribosome. For these experiments, cells depleted of putative 5S RNP biogenesis factors were pulse-labeled followed by a 6 hr chase to allow significant integration of the newly synthesized 5S rRNA into ribosomes. We also analyzed 5S integration into preribosomes in cells treated with the chemotherapeutic agents ActD or 5FU, which block rRNA

Cell PRESS Open ACCESS

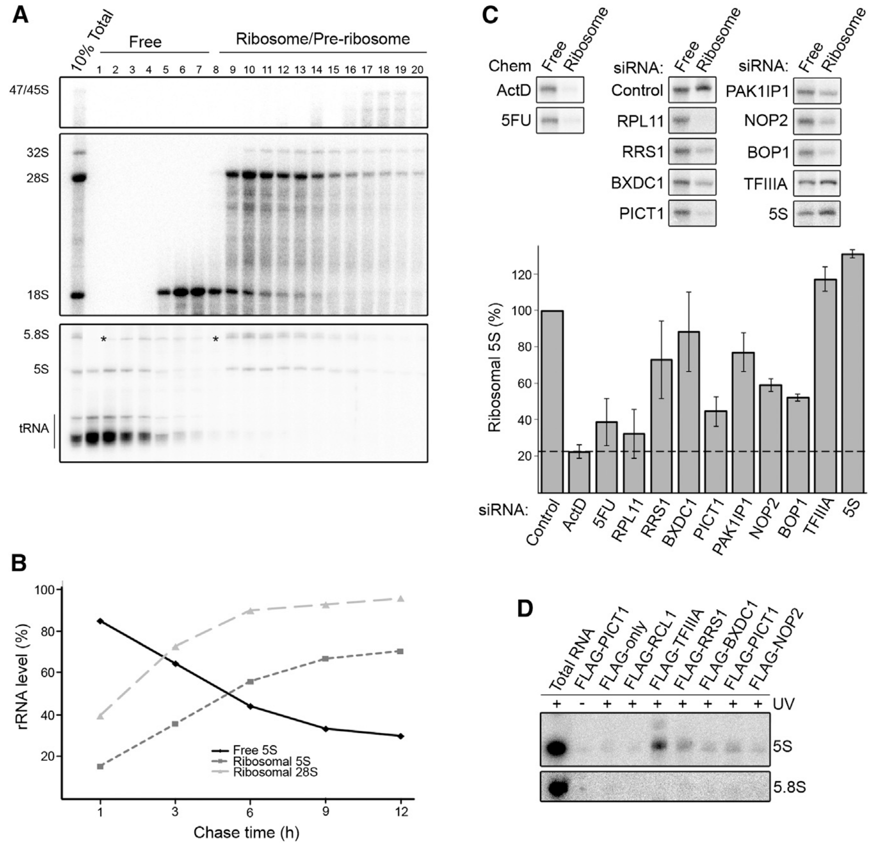

Figure 5. The Tumor Suppressor PICT1 Directly Contacts the 5S rRNA and Mediates 5S RNP Integration into the Ribosome

(A) HEK293 cells were pulse-labeled using ^{32}P orthophosphate then grown in unlabeled media for 1, 3, 6, 9, or 12 hr before harvesting. Whole cell extracts were separated by glycerol gradient centrifugation. RNA from each fraction was extracted and analyzed by agarose-glyoxal and acrylamide gel electrophoresis, and labeled RNA was visualized using a phosphorimager. (Upper panel: 1 hr; middle panel: 6 hr; lower panel: 6 hr).

(B) Levels of 5S and 28S rRNAs in free (pooled fractions 1–7) and ribosomal (pooled fractions 9–20) complexes were quantified at each time point.

(C) HEK293 cells transfected with siRNAs targeting various 5S/ribosome biogenesis factors or treated with chemotherapeutic chemicals were analyzed as in (A) using a 6 hr chase (upper panel). The proportion of 5S rRNA in ribosomal complexes (pooled fractions 9–20) was calculated from at least three experiments (including that shown) and is given graphically (lower panel). Error bars indicate SD. Dashed line represents a baseline (ActD treatment) to which other data can be compared.

(D) HEK293 cells expressing FLAG-tagged 5S biogenesis factors were UV crosslinked, and covalently bound RNA-protein complexes were purified. Copurified 5S and 5.8S rRNAs were analyzed by northern blotting.

See also Tables S1 and S2 and Figure S4.

transcription and processing, respectively. Extracts from these cells were separated by glycerol gradient centrifugation; the fractions representing the free and ribosomal complexes were pooled and the RNA content analyzed and quantified (Figures 5C and S4B).

The integration of the 5S rRNA into ribosomal complexes was reduced to about 20% of that seen in control cells after treatment with ActD. We defined this as the background level of newly synthesized 5S rRNA present in the ribosomal fractions in the absence of pre-rRNAs. Interestingly, treatment of cells with 5FU, which blocks pre-rRNA processing but does not affect the levels of the 47S, 45S, and 32S pre-rRNAs (Figure 3C), resulted in a significant decrease in 5S rRNA integration into preribosomes (Figure 5C), suggesting that one action of this inhibitor is to block 5S RNP integration into ribosomes.

Knockdown of RPL11 or PICT1 resulted in a very strong reduction of 5S rRNA incorporation into ribosomes, similar to that seen after treatment with ActD (Figure 5C). Depletion of either NOP2 or BOP1 also had a notable, but not as significant, effect on 5S rRNA integration into ribosomes. Reducing the levels of PAK1IP1, RRS1, or BXDC1 resulted in only a slight reduction of 5S rRNA integration. Although depleting these factors inhibits production of ribosomes, this resulted in similar, or even increased, levels of 32S pre-rRNA compared to control cells and no significant change in total ribosome levels (Figure 3B), demonstrating that formation of the large ribosomal subunit precursor into which the 5S RNP is integrated was not impaired. Furthermore, knockdown of RRS1, PICT1, or RPL11 had a similar effect on large ribosomal subunit production and 32S pre-rRNA accumulation, but PICT1 and RPL11 depletion had a significantly stronger influence on 5S rRNA integration into ribosomal complexes. Knockdown of RRS1 or BXDC1 had surprisingly little effect on 5S RNP recruitment into ribosomes considering that both proteins are required for 5S RNP integration into the large ribosomal subunit in yeast (Zhang et al., 2007). Interestingly, using siRNAs targeting TFIIIA or the 5S rRNA, which blocked ribosome production to a similar extent as depletion of PAK1IP1, resulted in a subtle but reproducible increase in 5S rRNA integration into ribosomes (Figure 5C). This is likely to reflect the fact that TFIIIA and 5S rRNA knockdowns affect ribosome biogenesis by reducing 5S rRNA synthesis, which would in turn decrease the free 5S rRNA pool leading to an increased uptake of newly made 5S RNP into the ribosome.

Our data show that RPL11, the putative tumor suppressor PICT1, and to a lesser extent BOP1 and NOP2 are important for the integration of the 5S RNP into the ribosome.

PICT1, RRS1, BXDC1, and TFIIIA Directly Contact the 5S rRNA

We have successfully identified factors that are required for 5S RNP localization (RRS1 and BXDC1) and integration into the ribosome (RPL11 and PICT1). It is possible that these proteins are directly involved in 5S RNP localization/incorporation, but these effects could also be indirect and arise due to impairment

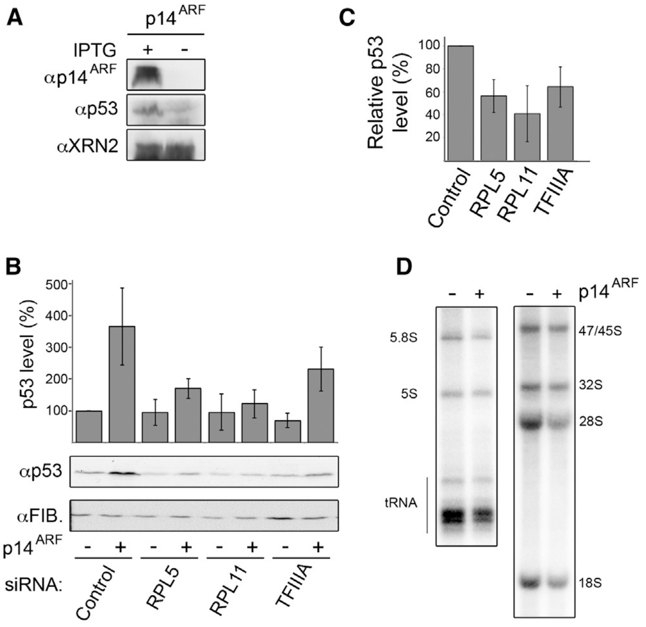

Figure 6. The 5S RNP Complex Is Required for p14^{ARF}-Mediated Activation of p53

(A) U2OS cells stably transfected with a plasmid expressing p14^{ARF} were untreated (–) or treated (+) with IPTG for 12 hr and then analyzed by western blotting.

(B) Expression of p14^{ARF} was induced (+) or not (–) in cells transfected with siRNAs targeting 5S RNP components. Levels of p53 in three replicate experiments were analyzed by western blotting and quantified. Error bars indicate SD.

(C) The relative difference between p53 induction in response to p14^{ARF} overexpression in cells transfected with control siRNAs and those targeting 5S RNP components was calculated based on the data shown in (B). Error bars indicate SD.

(D) Control U2OS cells or those expressing p14^{ARF} (as in [A]) were pulse-labeled using ^{32}P orthophosphate. RNA was analyzed by polyacrylamide and agarose-glyoxal gel electrophoresis and visualized using a phosphorimager.

See also Tables S1 and S3.

of other steps upstream of 5S RNP biogenesis. It is highly likely, however, that factors that directly contact the 5S RNP are functionally involved in 5S RNP recruitment or biogenesis. We therefore used the UV crosslinking and complex purification method, described earlier for RPL5 and MDM2 (Figure 1D), to identify biogenesis factors that bind the 5S rRNA. HEK293 cells stably expressing FLAG-tagged RRS1, BXDC1, PICT1, NOP2, and TFIIIA (Figure S4C) were irradiated with UV light and the proteins, and any covalently bound RNAs, isolated using a denaturing, two-step purification procedure. Coprecipitated rRNA was then analyzed by northern blotting. The 5S rRNA was crosslinked to PICT1, TFIIIA, RRS1, and BXDC1 indicating that these proteins directly contact this RNA (Figure 5D). Little or no 5S rRNA was coprecipitated with either the FLAG-tag alone, FLAG-RCL1 (a small subunit ribosome biogenesis factor), or when UV crosslinking was omitted. NOP2 did not reliably precipitate 5S rRNA above background levels.

These crosslinking data therefore confirm that PICT1 plays a direct role in 5S RNP recruitment into the ribosome and that RRS1 and BXDC1 regulate nucleolar localization of the 5S RNP by contacting the complex.

The 5S RNP Is Required for the Activation of p53 by the Tumor Suppressor p14^{ARF}

In addition to the 5S RNP-dependent pathway characterized above, p53 is also activated in response to many signals other than nucleolar stress. Interestingly, it has recently been shown that RPL11 is also important for the activation of p53 by the tumor suppressor p14^{ARF} following oncogenic stress (Dai et al., 2012), suggesting that the 5S RNP may coordinate p53 induction in response to multiple stresses. We therefore tested whether all the 5S RNP components are essential for the activity of p14^{ARF}. The levels of the 5S rRNA (via TFIIIA knockdown), RPL11, or RPL5 were reduced by RNAi in U2OS cells in which the expression of p14^{ARF} could be induced (Llanos et al., 2001), and the levels of p53 were then measured in the presence or absence of p14^{ARF} (Figures 6A and 6B).

In control cells, expression of p14^{ARF} resulted in increased levels of p53 as expected, but knockdown of RPL11 or RPL5 significantly reduced this response. The knockdown of TFIIIA, which reduced but did not abolish 5S rRNA transcription, also notably decreased p53 induction by p14^{ARF} overexpression suggesting that the 5S rRNA is also required for this response. Importantly, in each of three experiments performed, p53 induction was reduced relative to the control and appeared significant (Figure 6C). Our data therefore demonstrate that all the 5S RNP components are essential for the full activity of the tumor suppressor p14^{ARF}. Furthermore, expression of p14^{ARF} also inhibits ribosome biogenesis (Bertwistle et al., 2004; Itahana et al., 2003; Figure 6D). We propose that, in addition to directly inhibiting MDM2, p14^{ARF} also functions by increasing the amount of nonribosomal 5S RNP by blocking ribosome production. This highlights a central role for the 5S RNP in mediating crosstalk between different pathways of p53 activation.

DISCUSSION

We describe a mechanism by which ribosome biogenesis is directly coupled to cell proliferation, and we show that the abundance of the 5S RNP determines the basal levels of p53 in the cell. All three components of the 5S RNP, RPL5, RPL11, and the 5S rRNA, probably as a trimeric complex, are required for p53 activation in response to impaired ribosome production (Figure 7). After submission of our manuscript, the involvement of the 5S rRNA, as part of the 5S RNP, in the regulation of the MDM2-p53 checkpoint was also reported by the Thomas laboratory (Donati et al., 2013). Both papers come to the conclusion that the 5S RNP regulates p53 in response to changes in ribosome synthesis. Here, we also show that MDM2 directly binds the 5S rRNA in vivo and provide a detailed analysis of 5S RNP composition and dynamics. Furthermore, we define direct roles for PICT1 in 5S RNP integration into the ribosome and for RRS1 and BXDC1 in the nucleolar localization of 5S RNP components. Our data also indicate that p53 activation by the tumor suppressor p14^{ARF} involves the 5S RNP, suggesting crosstalk between the cellular responses to ribotoxic and oncogenic stress. Taken together, these data identify the 5S RNP as a key coordinator of

Cell PRESS
Open ACCESS

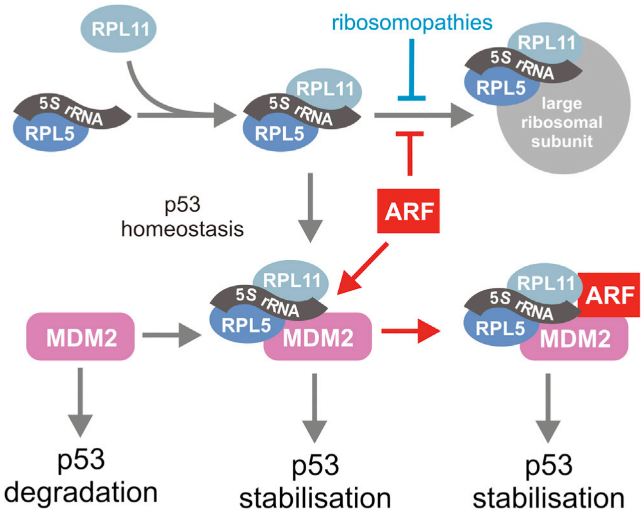

Figure 7. The 5S RNP Regulates p53 Homeostasis and Its Activation in Response to Nucleolar and p14ARF Overexpression
Schematic overview showing the central role of the 5S RNP in coupling the pathway of ribosome biogenesis to cellular proliferation.

the cellular responses to both pro- and antiproliferative signals (Figure 7).

The 5S RNP Interacts with MDM2 and Regulates p53

The 5S RNP accumulates in the nucleoplasm when ribosome biogenesis is blocked, where it associates with MDM2. Our data show that the 5S rRNA directly contacts MDM2 and that this interaction is increased when ribosome biogenesis is inhibited. Furthermore, the high level of the 5S rRNA/RPL5 complex (>200,000 copies per cell) in the nucleoplasm of normal cells is far in excess of most basic transcription factors indicating that any interaction between the dimeric 5S rRNA/RPL5 complex and MDM2 is unlikely to affect MDM2 activity. Therefore, the trimeric 5S RNP, rather than its individual components or subcomplexes, is the central regulator of MDM2 and therefore p53, which is consistent with earlier reports that both RPL5 and RPL11 are required for p53 activation in response to ActD treatment (Bursać et al., 2012; Fumagalli et al., 2012; Sun et al., 2010) and the recent report that the 5S rRNA is also required for this regulation (Donati et al., 2013). A 5S rRNA, RPL5, MDM2, p53, and 5.8S rRNA complex was previously described (Marechal et al., 1994). We do not find the 5.8S rRNA in any of our ‘‘nonribosomal’’ 5S RNP complexes, and the presence of this rRNA, which is not normally found outside the ribosome, may also indicate coprecipitation of the large ribosomal subunit in the results reported by Marechal et al. (1994).

Our data demonstrate that RPL5 is required for the accumulation and 3′ processing of the 5S rRNA, and it is likely that interaction with the RPL5/5S rRNA serves to stabilize RPL11. The abundance of the 5S rRNA/RPL5 complex in the cell, and the evidence that RPL11 is synthesized in excess (Lam et al., 2007), suggests that the binding of RPL11 to the 5S rRNA/RPL5 complex represents a key step in the p53 response and must be carefully regulated. In contrast to an earlier report (Bursać et al., 2012), but consistent with other observations (Chakraborty et al., 2011), we see RPL11 and RPL5 accumulate in the nucleoplasm when ribosome biogenesis is blocked, which may, however, reflect a difference in cell types used in the different studies.

Using an MDM2 mutant that has previously been demonstrated to prevent interaction with both RPL5 and RPL11 (Macias et al., 2010), we show that the direct interaction of the 5S rRNA and MDM2 is dependent on the presence of RPL5 and RPL11. Interestingly, using a different approach involving RNAi-mediated depletion of 5S RNP components, Donati et al. (2013) also demonstrate a mutually dependent interaction of the complex components with MDM2. Thus, the findings of the two papers are complementary and both come to the conclusion that the assembled 5S RNP regulates MDM2 and thereby p53.

Factors Involved in Biogenesis of the Large Ribosomal Subunit Regulate 5S RNP-Mediated Modulation of p53

Integration of the 5S RNP into the ribosome is essential for 28S and 5.8S rRNA processing, meaning that production of the large ribosomal subunit is closely coupled to the amount of free 5S RNP in the cell and consequently to p53 levels. We find that defects in ribosome production cause an increase in RPL11 levels in the nucleoplasm and p53 activation in a 5S RNP-dependent manner. Interestingly, our data demonstrate that the tumor suppressor PICT1, which directly binds to the 5S rRNA in vivo in our UV crosslinking experiment, is an essential ribosome biogenesis factor that is directly responsible for 5S RNP integration into the ribosome. This explains earlier observations that PICT1 directly binds RPL11 and RPL5 in vitro and is essential for retaining RPL11 in the nucleolus (Sasaki et al., 2011). NOP2 or BOP1 were also important for 5S RNP integration into the ribosome but to a lesser extent. These factors could directly modulate 5S RNP integration, potentially by bridging interactions between the 5S RNP and the preribosome, or alternatively be essential for upstream steps in the process.

Much to our surprise, and in contrast to the situation in yeast (Zhang et al., 2007), we found that neither RRS1 nor BXDC1 was associated with the 5S RNP outside the preribosome. Furthermore, depletion of either of these proteins had little or no impact on 5S RNP recruitment into the ribosome. It is, however, clear that these two proteins, which directly contact the 5S rRNA, have an important function in the nucleolar localization of RPL5 or both RPL5 and RPL11 in humans. Surprisingly, this reduction of RPL5 and/or RPL11 levels in the nucleolus has only a minor effect on 5S RNP integration into the ribosome.

The 5S RNP Is Essential for the Cellular Response to p14ARF Overexpression

RPL11 has been shown to bind p14ARF and form an RPL11/p14ARF/MDM2/p53 complex that is required for complete p14ARF-dependent suppression of MDM2 and activation of p53 (Dai et al., 2012). We show here that RPL5 and the 5S rRNA, probably as part of the assembled 5S RNP complex, are also important for this process (Figure 7). This supports results where a mutation (C305F) in the zinc finger of MDM2, which cannot bind RPL11 and RPL5, accelerates Myc-induced lymphomagenesis in mice (Macias et al., 2010). It is well established that p14ARF

induction inhibits ribosome biogenesis, primarily targeting the production of the large ribosomal subunit (Figure 6). These data imply that p14^{ARF} predominantly functions by impeding ribosome biogenesis, causing an increase in the levels of nonribosomal 5S RNP (Figure 7). The 5S RNP, together with p14^{ARF}, would then inhibit MDM2 leading to p53 activation.

Regulation of p53 Homeostasis and Cellular Proliferation by the 5S RNP

We have clearly shown that the amount of the nonribosomal 5S RNP in the cell determines the basal level of the tumor suppressor p53. This provides a means by which the rate of ribosome biogenesis is coupled to p53 homeostasis and therefore to cellular proliferation (Figure 7). However, Donati et al. (2013) observe only a slight reduction in p53 levels upon depletion of 5S RNP components. This may reflect differences in the efficiency or duration of siRNA-mediated knockdown of the various components or the different siRNA treatment times. Differences between transfection efficiencies may also explain why we observed a clear defect on 5S rRNA levels and pre-rRNA processing using the 5S rRNA-specific siRNA that was not observed in the other publication (Donati et al., 2013). The 5S RNP is a combined product of RNA pol II and RNA pol III, and the levels of free 5S RNP in the cell directly relate to the processing of the RNA-pol-I-transcribed rRNAs. The 5S RNP is therefore an ideal marker to coordinate gene expression from all three RNA polymerases and the rate of ribosome biogenesis, itself an essential aspect of cellular growth. Furthermore, RPL11 has previously been shown to regulate the proproliferative factor, c-Myc (Dai et al., 2007). We would suggest that c-Myc regulation is mediated by the 5S RNP, and we therefore propose this complex to be a critical regulator of several cellular signaling pathways.

A number of ribosomopathies, including Diamond Blackfan anemia, 5q syndrome, and Treacher Collins syndrome, all result in misregulation of p53, probably due to impaired ribosome production increasing the amount of nonribosomal 5S RNP (Figure 7; Fumagalli and Thomas, 2011). The increased levels of p53 cause apoptosis during facial development in Treacher Collins syndrome (Jones et al., 2008). In Diamond Blackfan anemia and 5q syndrome, anemia is predicted to result from an inability to meet the increased demand for ribosomes during erythropoiesis (Boultwood et al., 2012). Paradoxically, many of these diseases also result in an increased propensity for cancer (Freed et al., 2010; Narla and Ebert, 2010). However, it is possible that either the p53 response becomes desensitized or that the cells adapt to high p53 levels. Our data, therefore, highlight the 5S RNP as a future target for anticancer and antiribosomopathy drugs.

EXPERIMENTAL PROCEDURES

RNAi and the Analysis of rRNA Processing

HEK293 cell, U2OS cells, or U2OS cells expressing p14^{ARF} under the control of an isopropyl β-D-1-thiogalactopyranoside (IPTG)-inducible promoter (Llanos et al., 2001) were transfected with siRNAs (see Table S1) using RNAiMAX Lipofectamine. Harvested cells were analyzed by northern or western blotting (see Tables S2 and S3). For pulse-labeling experiments, cells were treated with siRNAs or chemotherapeutic agents (ActD and 5FU) and then pulse-labeled with ^{32}P orthophosphate (Sloan et al., 2013). RNAs extracted from the cells were analyzed by agarose-glyoxal and denaturing-polyacrylamide gel electrophoresis.

Immunoprecipitation and Glycerol Gradient Analysis

Whole cell extracts were separated on 10%–40% glycerol gradients, and the resultant fractions analyzed by gel electrophoresis followed by western/northern blotting or, in the case of labeled RNAs, phosphorimager analysis. Immunoprecipitation reactions were performed with whole cell extracts or pooled gradient fractions using either an anti-FLAG antibody or anti-MDM2 antibodies. Coprecipitated RNAs were extracted and analyzed by northern blotting.

Immunofluorescence

HEK293 cell lines stably expressing inducible FLAG-tagged RPL5 or RPL11 were treated with ActD or specific siRNAs and then analyzed by immunofluorescence using anti-FLAG and anti-fibrillarin antibodies. Images were captured with a Zeiss Axiovert 200M inverted microscope.

UV Crosslinking of 5S rRNA and 5S RNP Biogenesis Factors

HEK293 cells expressing FLAG/His-tagged proteins of interest were UV irradiated. Complexes were purified using anti-FLAG antibodies, eluted using PreScission Protease, and then purified using nickel-nitrilotriacetic acid (NTA) under denaturing conditions (6 M guanidium-HCl). Coprecipitated RNAs were eluted using Proteinase K and analyzed by northern blotting.

See Extended Experimental Procedures for more information.

SUPPLEMENTAL INFORMATION

Supplemental Information includes Extended Experimental Procedures, four figures, and three tables and can be found with this article online at http://dx.doi.org/10.1016/j.celrep.2013.08.049.

ACKNOWLEDGMENTS

We would like to thank Neil Perkins for providing the inducible p14^{ARF} U2OS cell line and anti-p14^{ARF} antibody. We would also like to thank Neil Perkins, Robert Lightowlers, and Claudia Schneider for critically reading the manuscript. This work was funded by the Wellcome Trust (N.J.W.), a European Molecular Biology Organization short-term fellowship (K.E.S.), and the Deutsche Forschungsgemeinschaft (BO 3442/1-1; to M.T.B.).

Received: June 30, 2013
Revised: August 16, 2013
Accepted: August 30, 2013
Published: October 10, 2013

REFERENCES

Bertwistle, D., Sugimoto, M., and Sherr, C.J. (2004). Physical and functional interactions of the Arf tumor suppressor protein with nucleophosmin/B23. Mol. Cell. Biol. *24*, 985–996.

Boultwood, J., Pellagatti, A., and Wainscoat, J.S. (2012). Haploinsufficiency of ribosomal proteins and p53 activation in anemia: Diamond-Blackfan anemia and the 5q- syndrome. Adv. Biol. Regul. *52*, 196–203.

Bursać, S., Brdovčak, M.C., Pfannkuchen, M., Orsolić, I., Golomb, L., Zhu, Y., Katz, C., Daftuar, L., Grabušić, K., Vukelić, I., et al. (2012). Mutual protection of ribosomal proteins L5 and L11 from degradation is essential for p53 activation upon ribosomal biogenesis stress. Proc. Natl. Acad. Sci. USA *109*, 20467–20472.

Chakraborty, A., Uechi, T., and Kenmochi, N. (2011). Guarding the 'translation apparatus': defective ribosome biogenesis and the p53 signaling pathway. Wiley Interdiscip. Rev. RNA *2*, 507–522.

Ciganda, M., and Williams, N. (2011). Eukaryotic 5S rRNA biogenesis. Wiley Interdiscip. Rev. RNA *2*, 523–533.

Dai, M.S., Arnold, H., Sun, X.X., Sears, R., and Lu, H. (2007). Inhibition of c-Myc activity by ribosomal protein L11. EMBO J. *26*, 3332–3345.

Dai, M.S., Challagundla, K.B., Sun, X.X., Palam, L.R., Zeng, S.X., Wek, R.C., and Lu, H. (2012). Physical and functional interaction between ribosomal protein L11 and the tumor suppressor ARF. J. Biol. Chem. *287*, 17120–17129.

Donati, G., Peddigari, S., Mercer, C.A., and Thomas, G. (2013). 5S ribosomal RNA is an essential component of a nascent ribosomal precursor complex that regulates the Hdm2-p53 checkpoint. Cell Rep. *4*, 87–98.

Elenbaas, B., Dobbelstein, M., Roth, J., Shenk, T., and Levine, A.J. (1996). The MDM2 oncoprotein binds specifically to RNA through its RING finger domain. Mol. Med. *2*, 439–451.

Freed, E.F., Bleichert, F., Dutca, L.M., and Baserga, S.J. (2010). When ribosomes go bad: diseases of ribosome biogenesis. Mol. Biosyst. *6*, 481–493.

Fumagalli, S., and Thomas, G. (2011). The role of p53 in ribosomopathies. Semin. Hematol. *48*, 97–105.

Fumagalli, S., Ivanenkov, V.V., Teng, T., and Thomas, G. (2012). Suprainduction of p53 by disruption of 40S and 60S ribosome biogenesis leads to the activation of a novel G2/M checkpoint. Genes Dev. *26*, 1028–1040.

Hölzel, M., Rohrmoser, M., Schlee, M., Grimm, T., Harasim, T., Malamoussi, A., Gruber-Eber, A., Kremmer, E., Hiddemann, W., Bornkamm, G.W., and Eick, D. (2005). Mammalian WDR12 is a novel member of the Pes1-Bop1 complex and is required for ribosome biogenesis and cell proliferation. J. Cell Biol. *170*, 367–378.

Horn, H.F., and Vousden, K.H. (2008). Cooperation between the ribosomal proteins L5 and L11 in the p53 pathway. Oncogene *27*, 5774–5784.

Itahana, K., Bhat, K.P., Jin, A., Itahana, Y., Hawke, D., Kobayashi, R., and Zhang, Y. (2003). Tumor suppressor ARF degrades B23, a nucleolar protein involved in ribosome biogenesis and cell proliferation. Mol. Cell *12*, 1151–1164.

Jones, N.C., Lynn, M.L., Gaudenz, K., Sakai, D., Aoto, K., Rey, J.P., Glynn, E.F., Ellington, L., Du, C., Dixon, J., et al. (2008). Prevention of the neurocristopathy Treacher Collins syndrome through inhibition of p53 function. Nat. Med. *14*, 125–133.

Kim, J.Y., Seok, K.O., Kim, Y.J., Bae, W.K., Lee, S., and Park, J.H. (2011). Involvement of GLTSCR2 in the DNA Damage Response. Am. J. Pathol. *179*, 1257–1264.

Lam, Y.W., Lamond, A.I., Mann, M., and Andersen, J.S. (2007). Analysis of nucleolar protein dynamics reveals the nuclear degradation of ribosomal proteins. Curr. Biol. *17*, 749–760.

Li, M., and Gu, W. (2011). A critical role for noncoding 5S rRNA in regulating Mdmx stability. Mol. Cell *43*, 1023–1032.

Llanos, S., Clark, P.A., Rowe, J., and Peters, G. (2001). Stabilization of p53 by p14ARF without relocation of MDM2 to the nucleolus. Nat. Cell Biol. *3*, 445–452.

Macias, E., Jin, A., Deisenroth, C., Bhat, K., Mao, H., Lindström, M.S., and Zhang, Y. (2010). An ARF-independent c-MYC-activated tumor suppression pathway mediated by ribosomal protein-Mdm2 Interaction. Cancer Cell *18*, 231–243.

Marechal, V., Elenbaas, B., Piette, J., Nicolas, J.C., and Levine, A.J. (1994). The ribosomal L5 protein is associated with mdm-2 and mdm-2-p53 complexes. Mol. Cell. Biol. *14*, 7414–7420.

Narla, A., and Ebert, B.L. (2010). Ribosomopathies: human disorders of ribosome dysfunction. Blood *115*, 3196–3205.

Pan, W., Issaq, S., and Zhang, Y. (2011). The in vivo role of the RP-Mdm2-p53 pathway in signaling oncogenic stress induced by pRb inactivation and Ras overexpression. PLoS ONE *6*, e21625.

Reddy, R., and Busch, H. (1988). Small Nuclear RNAs: RNA Sequences, Structure, and Modifications. In Structure and Function of Major and Minor Small Nuclear Ribonucleoprotein Particles, M.L. Birnstiel, ed. (Berlin: Springer-Verlag), pp. 1–37.

Sasaki, M., Kawahara, K., Nishio, M., Mimori, K., Kogo, R., Hamada, K., Itoh, B., Wang, J., Komatsu, Y., Yang, Y.R., et al. (2011). Regulation of the MDM2-P53 pathway and tumor growth by PICT1 via nucleolar RPL11. Nat. Med. *17*, 944–951.

Sloan, K.E., Mattijssen, S., Lebaron, S., Tollervey, D., Pruijn, G.J., and Watkins, N.J. (2013). Both endonucleolytic and exonucleolytic cleavage mediate ITS1 removal during human ribosomal RNA processing. J. Cell Biol. *200*, 577–588.

Strezoska, Z., Pestov, D.G., and Lau, L.F. (2000). Bop1 is a mouse WD40 repeat nucleolar protein involved in 28S and 5. 8S RRNA processing and 60S ribosome biogenesis. Mol. Cell. Biol. *20*, 5516–5528.

Stumpf, C.R., and Ruggero, D. (2011). The cancerous translation apparatus. Curr. Opin. Genet. Dev. *21*, 474–483.

Sun, X.X., Wang, Y.G., Xirodimas, D.P., and Dai, M.S. (2010). Perturbation of 60 S ribosomal biogenesis results in ribosomal protein L5- and L11-dependent p53 activation. J. Biol. Chem. *285*, 25812–25821.

Talkish, J., Zhang, J., Jakovljevic, J., Horsey, E.W., and Woolford, J.L., Jr. (2012). Hierarchical recruitment into nascent ribosomes of assembly factors required for 27SB pre-rRNA processing in Saccharomyces cerevisiae. Nucleic Acids Res. *40*, 8646–8661.

Watkins, N.J., Lemm, I., Ingelfinger, D., Schneider, C., Hossbach, M., Urlaub, H., and Lührmann, R. (2004). Assembly and maturation of the U3 snoRNP in the nucleoplasm in a large dynamic multiprotein complex. Mol. Cell *16*, 789–798.

Zhang, J., Harnpicharnchai, P., Jakovljevic, J., Tang, L., Guo, Y., Oeffinger, M., Rout, M.P., Hiley, S.L., Hughes, T., and Woolford, J.L., Jr. (2007). Assembly factors Rpf2 and Rrs1 recruit 5S rRNA and ribosomal proteins rpL5 and rpL11 into nascent ribosomes. Genes Dev. *21*, 2580–2592.

Supplemental Information

EXTENDED EXPERIMENTAL PROCEDURES

Cell Culture and RNAi

HEK293 cells were stably transfected with plasmids to enable tetracycline-inducible expression of FLAG-RPL5, -RPL11, -MDM2 or putative 5S biogenesis factors. U2OS cells in which $p14^{ARF}$ expression could be induced by addition of 1 mM IPTG for 12 hr were kindly provided by Neil Perkins (Newcastle University) (Llanos et al., 2001). HEK293 or U2OS cells were transfected with siRNAs (listed in Table S1) using RNAiMAX lipofectamine (Invitrogen) for 48 hr before pulse labeling or 60 hr before harvesting for western blotting.

Analysis of Pre-rRNA Processing

For pulse-labeling experiments, cells depleted of ribosome biogenesis factors by RNAi (48 hr) or treated with chemotherapeutic agents (Actinomycin D (5 nM) or 5-fluorouracil (50 μM) for 10 hr) were grown in phosphate-free DMEM for 1 hr, followed by phosphate-free DMEM containing 15 μCi/ml ^{32}P labeled inorganic orthophosphate for 1 hr. Cells were then grown in normal DMEM media for 3 hr. Cells were harvested, RNA was extracted using TRI-reagent (Sigma) and analyzed by agarose-glyoxal and denaturing polyacrylamide gel electrophoresis. Results were visualized using a phosphorimager (Typhoon FLA9500).

Extract Preparation, Immunoprecipitation, and Glycerol Gradient Analysis

HeLa cell nucleoplasmic and cytoplasmic extracts were kindly provided by Berthold Kastner and Reinhard Lührmann (MPI Goettingen). Whole cell extracts for immunoprecipitations or gradient analysis were prepared from HEK293 cells by sonication as in (Turner et al., 2009). Immunoprecipitation reactions were performed as in (Turner et al., 2012) using either an anti-FLAG antibody (Sigma, M2) or anti-MDM2 (Calbiochem, OP46). Co-precipitated RNAs were extracted and analyzed by northern blotting using an oligonucleotide probe to detect 5S or 5.8S rRNAs (see Table S2).

Whole cell extracts were separated on 10%–40% glycerol gradients by centrifugation at 52,000 rpm in an SW60 rotor for 90 min (Turner et al., 2009). Fractions were taken and used directly for western blotting (using antibodies listed in Table S3). Alternatively, RNA was extracted using phenol:chloroform:isoamylalcohol, ethanol precipitated and analyzed either by agarose-glyoxal or denaturing polyacrylamide gel electrophoresis as required.

5S Integration Assay

Control HEK293 cells, or cell depleted of putative 5S biogenesis factors or treated with chemotherapeutic agents (described in previous section) were pulse-labeled as described above, then grown in unlabeled media for 6 hr. Cells were harvested, whole cells extracts were prepared and separated by glycerol gradient centrifugation (as above). Fractions containing free complexes (1-7) and ribosomal complexes (9-20) were pooled and RNA was extracted and analyzed by denaturing polyacrylamide gel and agarose-glyoxal gel electrophoresis. Labeled RNAs were detected using a phosphorimager and the 5S rRNA levels in each of the pools were quantified using the ImageQuant software (GE Healthcare).

Immunofluorescence

For ActD treatment, HEK293 cell lines stably expressing FLAG-tagged RPL5 or RPL11 were treated with ActD and induced with 1 μg/ml tetracycline for 12 hr. For knockdowns, the cells were treated with siRNAs for 60 hr with 1 μg/ml tetracycline added for the final 12 hr. Immunofluorescence was performed as described previously (Turner et al., 2012). Cell were fixed with PBS containing 4% paraformaldehyde, permeabilised using PBS containing 0.2% Triton X-100 and then stained using anti-FLAG (SIGMA; rabbit) and anti-fibrillarin (72B9; mouse) antibodies and DAPI. Coverslips were mounted in Moviol and images were captured with a Zeiss Axiovert 200M inverted microscope with a Plan-Apochromat, 100 x / 1.40 oil DIC x / 0.17 objective, an AxioCam HRm camera and using Axiovision software.

UV Crosslinking of 5S rRNA and 5S RNP Biogenesis Factors

HEK293 cells expressing FLAG-His-tagged proteins of interest or the FLAG-His-tag alone were induced using 1 μg/ml tetracycline for 36 hr. Cells were washed with PBS and UV cross-linking (800 J/cm^2) was carried out using a Stratalinker (Stratagene). Cells were harvested in buffer containing 50 mM Tris/HCl (pH 7.6), 150 mM NaCl, 0.1% NP-40, 5 mM β-mercaptoethanol and protease inhibitors, lysed by sonication and extracts were clarified by centrifugation. Complexes were purified using anti-FLAG magnetic beads and eluted using PreScission Protease. A second purification step using Nickel-NTA was performed under denaturing conditions (6 M guanidium-HCL) and co-precipitated RNAs were eluted by Proteinase K digestion. RNAs were analyzed by northern blotting using probes hybridizing to 5S and 5.8S rRNA.

SUPPLEMENTAL REFERENCES

Elbashir, S.M., Harborth, J., Weber, K., and Tuschl, T. (2002). Analysis of gene function in somatic mammalian cells using small interfering RNAs. Methods *26*, 199–213.

Gambe, A.E., Matsunaga, S., Takata, H., Ono-Maniwa, R., Baba, A., Uchiyama, S., and Fukui, K. (2009). A nucleolar protein RRS1 contributes to chromosome congression. FEBS Lett. *583*, 1951–1956.

Kuroda, T., Murayama, A., Katagiri, N., Ohta, Y.M., Fujita, E., Masumoto, H., Ema, M., Takahashi, S., Kimura, K., and Yanagisawa, J. (2011). RNA content in the nucleolus alters p53 acetylation via MYBBP1A. EMBO J. *30*, 1054–1066.

Lee, S., Kim, J.Y., Kim, Y.J., Seok, K.O., Kim, J.H., Chang, Y.J., Kang, H.Y., and Park, J.H. (2012). Nucleolar protein GLTSCR2 stabilizes p53 in response to ribosomal stresses. Cell Death Differ. *19*, 1613–1622.

Rohrmoser, M., Hölzel, M., Grimm, T., Malamoussi, A., Harasim, T., Orban, M., Pfisterer, I., Gruber-Eber, A., Kremmer, E., and Eick, D. (2007). Interdependence of Pes1, Bop1, and WDR12 controls nucleolar localization and assembly of the PeBoW complex required for maturation of the 60S ribosomal subunit. Mol. Cell. Biol. *27*, 3682–3694.

Turner, A.J., Knox, A.A., Prieto, J.L., McStay, B., and Watkins, N.J. (2009). A novel small-subunit processome assembly intermediate that contains the U3 snoRNP, nucleolin, RRP5, and DBP4. Mol. Cell. Biol. *29*, 3007–3017.

Turner, A.J., Knox, A.A., and Watkins, N.J. (2012). Nucleolar disruption leads to the spatial separation of key 18S rRNA processing factors. RNA Biol. *9*, 175–186.

Cell PRESS Open ACCESS

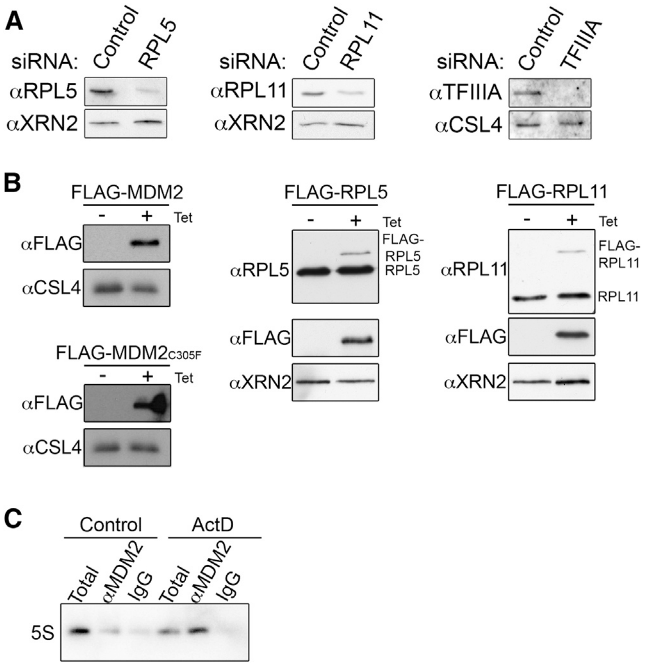

Figure S1. Analysis of the 5S RNP and Its Association with MDM2, Related to Figure 1

(A) Cells transfected with either control siRNAs or those targeting RPL5, RPL11 or TFIIIA were harvested 60 hr after transfection and proteins were analyzed by western blotting using antibodies raised against the targeted protein and either CSL4 or XRN2 as loading controls.

(B) HEK293 cells stably transfected with plasmids enabling expression of FLAG-tagged RPL5, RPL11, MDM2 or $MDM2_{C305F}$ were induced (+) with 1 μg/ml tetracycline or left uninduced (-) for 48 hr before harvesting. Proteins were analyzed by western blotting using the antibodies indicated.

(C) Immunoprecipitation, using an antibody that recognizes endogenous MDM2, was performed on extracts from U2OS cells either untreated (control) or treated with ActD. Co-precipitated RNAs were extracted and analyzed by northern blotting using a probe hybridizing to the 5S rRNA.

Cell PRESS
Open ACCESS

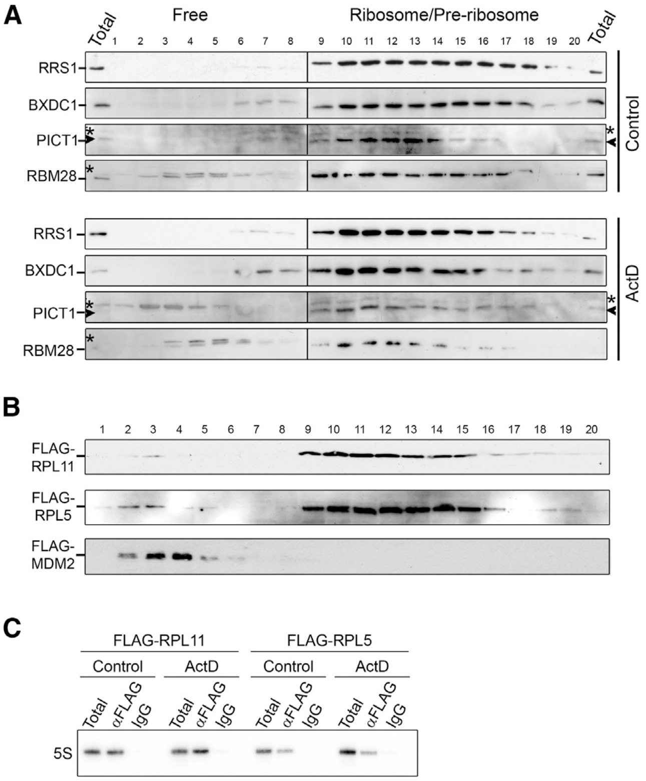

Figure S2. Characterization of the 5S RNP, Related to Figure 2

(A) Extracts from HEK293 cells either treated with ActD or untreated (control) were analyzed by glycerol gradient centrifugation followed by western blotting using the antibodies indicated to the left of each panel. * denotes non-specific proteins that cross-react with anti-PICT1 and anti-RBM28 antibodies. Arrowhead indicates specific PICT1 signal.

(B) Expression of FLAG-RPL5, FLAG-RPL11 or FLAG-MDM2 was induced in HEK293 stable cell lines by addition of 1 μg/ml tetracycline for 48 hr. Cells were harvested and extracts were separated by glycerol gradient centrifugation. Proteins were analyzed by western blotting using an anti-FLAG antibody.

(C) HEK293 cells stably expressing FLAG-RPL5 and FLAG-RPL11, induced for 48 hr, were treated with ActD for 10 hr. Whole cell extracts were prepared, separated by glycerol gradient centrifugation and fractions 1-7 containing free complexes were pooled. The pooled fractions were analyzed by immunoprecipitation using an anti-FLAG antibody. RNA was extracted and analyzed by northern blotting using a probe against the 5S rRNA.

Cell PRESS
Open ACCESS

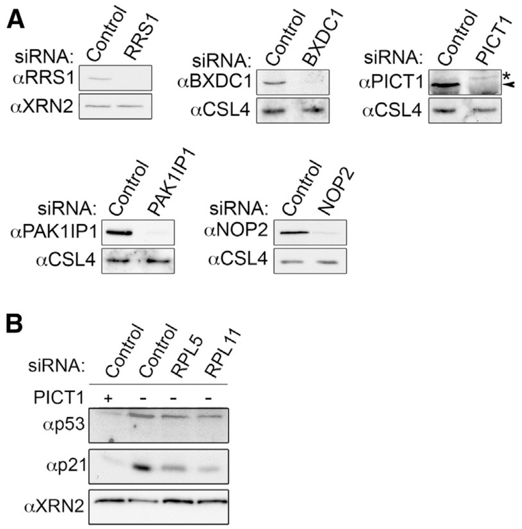

Figure S3. Depletion of Large Ribosomal Subunit Biogenesis Factors Causes Pre-rRNA Processing Defects and Activates p53 through the 5S RNP/MDM2 Pathway, Related to Figure 3

(A) HEK293 cells were transfected with either control siRNAs or those targeting LSU/5S biogenesis factors. Cells were harvested 60 hr after transfection and proteins were analyzed by western blotting using antibodies raised against the targeted protein and either CSL4 or XRN2 as loading controls. * indicates non-specific protein detected by anti-PICT1 antibody while PICT1 is marked with arrowhead.

(B) U2OS cells were transfected with either control siRNAs or those targeting RPL5 or RPL11, individually or in combination with those to deplete PICT1. 60 hr after the knockdown, cells were harvested and proteins analyzed by western blotting using antibodies to detect p53, the p53-activated protein, p21, or XRN2, as a loading control.

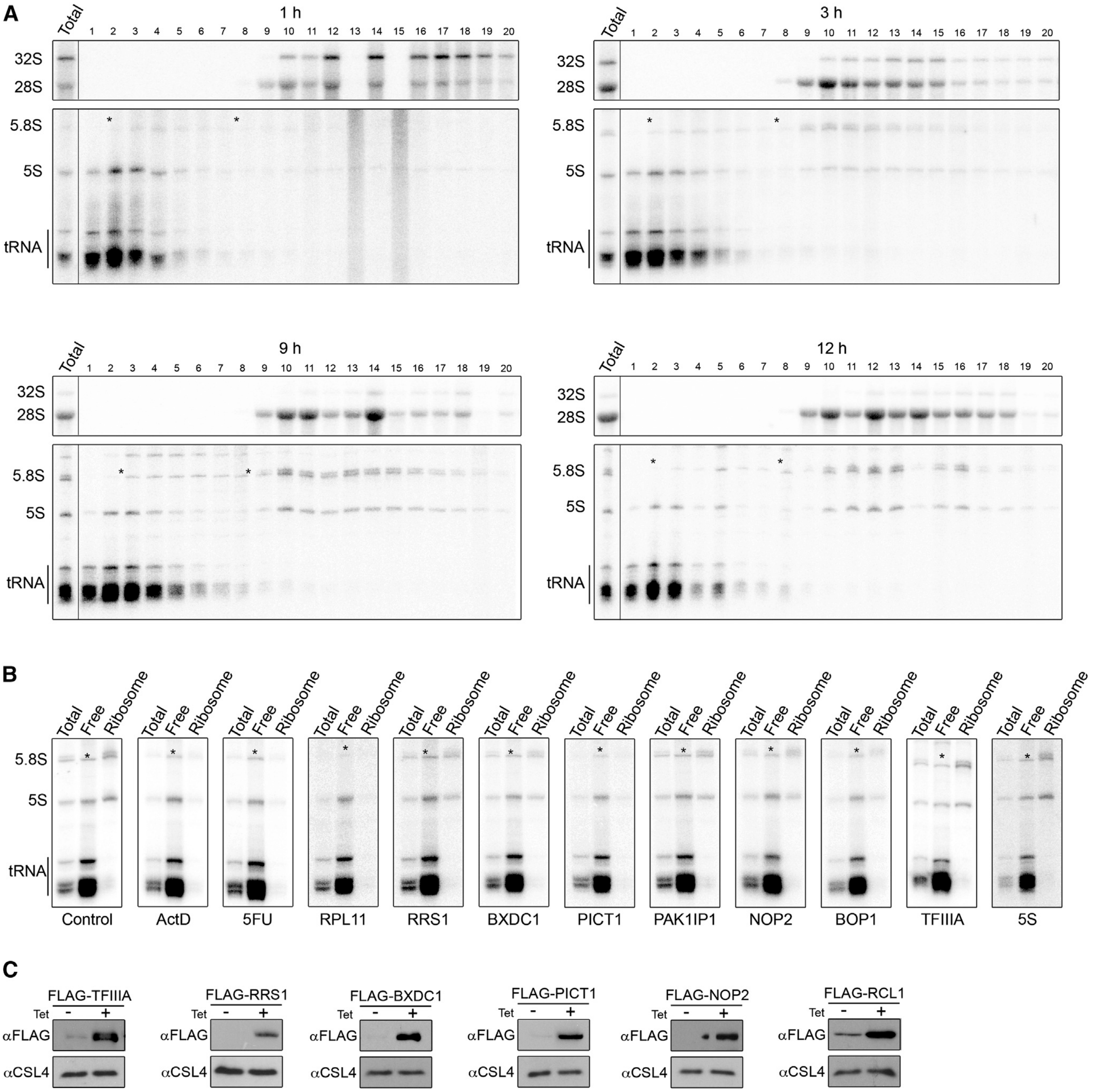

Figure S4. Factors Required for Integration of the 5S RNP into the Ribosome, Related to Figure 5

(A) HEK293 cells were pulse-labeled with ^{32}P orthophosphate and then grown in normal media and harvested at various time points during a 12 hr period. Whole cell extracts were prepared and separated by glycerol gradient centrifugation. RNA was extracted and analyzed by denaturing polyacrylamide and agarose-glyoxal gel electrophoresis. RNA was detected using a phosphorimager.

(B) HEK293 cells were depleted of 5S biogenesis factors or treated with chemotherapeutic chemicals and then pulse-labeled using ^{32}P orthophosphate. Cells were then grown in unlabeled media for 6 hr before harvesting. Complexes were separated by glycerol gradient centrifugation as in (A) and fractions 1-7 and fractions 9-20, which contain the free and ribosomal complexes, respectively, were pooled. RNAs were analyzed on a denaturing polyacrylamide gel and labeled RNAs were detected using a phosphorimager. * denotes a labeled RNA (probably U1 snRNA) of the same size as 5.8S rRNA that is present in non-ribosomal fractions. Note that this RNA is not affected by ActD treatment confirming that it is not 5.8S rRNA. Furthermore, 5.8S rRNA is not found outside ribosomal complexes and not detected in these fractions by northern blotting (Figure S1A).

(C) HEK293 cells stably transfected with plasmids to enable expression of FLAG-tagged 5S biogenesis factors were untreated (-) or treated with 1 μg/ml tetracycline (+) to induce tagged protein expression. Protein samples were analyzed by western blotting using both an anti-FLAG antibody and as a loading control, anti-CSL4.
